# How do students learn to move? A systematic review of collaborative and game-based pedagogies in physical education

**DOI:** 10.3389/fspor.2026.1830716

**Published:** 2026-07-02

**Authors:** Sohom Saha, İsmail İlbak, Ellie Abdi, Joe Deutsch, Borko Katanić, Suchishrava Choudhary, Prashant Kumar Choudhary, Larissa Nayara de Souza, Roque Ribeiro da Silva Júnior, Gusztáv József Tornóczky

**Affiliations:** 1 Department of Sport Psychology, Lakshmibai National Institute of Physical Education, Gwalior, Madhya Pradesh, India; 2 Faculty of Sports Science, İnönü University, Malatya, Türkiye; 3 Teaching & Learning- Kinesiology, Department, Montclair State University, Montclair, NJ, United States; 4 Health, Nutrition, & Exercise Science, Department, North Dakota State University, Fargo, ND, United States; 5 Montenegrin Sports Academy, Podgorica, Montenegro; 6 Department of Physical Education Pedagogy, Lakshmibai National Institute of Physical Education, Gwalior, Madhya Pradesh, India; 7 State University of Rio Grande do Norte (UERN), Grande do Norte, Brazil; 8 Physical Education and Exercise Centre, Medical School, University of Pécs, Pécs, Hungary; 9 Faculty of Education and Psychology, Institute of Health Promotion and Sport Sciences, ELTE Eötvös Loránd University, Budapest, Hungary

**Keywords:** collaborative learning, game-based learning, motor skill development, movement competence, physical education

## Abstract

**Background:**

Collaborative and game-based pedagogical approaches are increasingly applied in physical education (PE) to enhance engagement and support meaningful movement learning. However, empirical evidence remains fragmented across educational levels, instructional models, outcome domains, and methodological designs, limiting coherent synthesis and interpretation. This systematic review followed the PRISMA 2020 guide-lines and was registered in PROSPERO (no. CRD420261306696). It aimed to map quasiexperimental evidence on collaborative and game-based pedagogies implemented in formal Physical Education contexts, as well as to synthesize their associations with movement-related outcomes.

**Methods:**

A systematic search of Scopus, Web of Science, PubMed, ERIC, and SPORTDiscus was conducted for studies published between January 2011 and December 2025. Seventeen peer-reviewed studies met predefined PICOS-based inclusion criteria. Eligible studies implemented collaborative or game-based instructional models within PE or Physical Education Teacher Education contexts and assessed movement-related outcomes. Data were synthesised using narrative synthesis supported by structured descriptive evidence mapping. Due to substantial heterogeneity in study designs, participant characteristics, pedagogical approaches, and outcome measures, quantitative meta-analysis was not considered appropriate. Methodological quality was appraised using an adapted Joanna Briggs Institute checklist.

**Results:**

Seventeen quasi-experimental studies were included, involving preschool (*n* = 1), primary (*n* = 4), secondary (*n* = 4), and university/PETE populations (*n* = 8). Pedagogical models included cooperative learning, peer teaching, Team Games Tournament, exergame-based learning, and structured game-based movement models (*n* = 3 each), while tactical or games-based approaches were less frequently examined (*n* = 2). Sport-specific technical skills were the most commonly assessed outcomes (35.3%), followed by fundamental movement skills, motor competence, game performance, and psychosocial/fitness-related outcomes. Motor or game-performance improvements were reported in 41.2% of studies, mixed effects in 29.4%, and no superior motor learning in 29.4%, with no negative outcomes reported.

**Discussion:**

Collaborative and game-based pedagogies appear pedagogically viable in PE, particularly when instructional design aligns with targeted movement outcomes. However, heterogeneity in study design, outcome measurement, and participant age limits comparative inference. Future research should prioritise adolescent populations, longitudinal designs, and ecologically valid movement assessments.

**Systematic Review Registration:**

https://www.crd.york.ac.uk/PROSPERO/view/CRD420261306696.

## 1 Introduction

Physical education plays a foundational role in promoting movement competence, motor skill development, physical literacy, and lifelong engagement in physical activity across childhood, adolescence, and early adulthood (1, 2). Engagement in sport and physical activity extends beyond physical conditioning, acting as a transformative process that simultaneously enhances physiological health, psychological resilience, and overall well-being (3, 4). The acquisition of fundamental movement skills, sport-specific techniques, and coordinated movement patterns supports athletic performance and also contributes to physical confidence, health-behaviour formation, and broader psychosocial development (1). Contemporary physical education curricula increasingly emphasise learner-centred, active learning experiences (e.g., engagement, problem-solving, and social interaction) rather than passive skill repetition, and within this shift, collaborative learning and game-based instructional models (e.g., Tactical Games Model) have gained prominence for integrating cognitive, social, and motor learning in authentic movement contexts (2, 5).

Collaborative learning approaches, including cooperative learning, peer teaching, and team-based instructional structures, promote shared responsibility, reciprocal feedback, and collective problem-solving (6). Evidence from school and university settings demonstrates that peer-mediated instruction enhances motor accuracy, task understanding, and feedback quality in sports such as tennis and volleyball (7, 8). Cooperative learning models have also been shown to improve basketball skill execution and cognitive engagement among elementary and secondary students (9, 10). These pedagogical structures foster meaningful practice opportunities, increased on-task behaviour, and social accountability, which collectively support motor learning processes (11). At the same time, collaborative environments can enhance learner motivation and engagement, reinforcing sustained participation and deliberate practice (12).

Game-based learning further complements collaborative pedagogy by situating skill acquisition within dynamic, representative performance environments. Tactical game approaches emphasize decision-making, perceptual awareness, and contextual problem-solving rather than isolated technical drills. University-based interventions using the Tactical Games Model have demonstrated improvements in basketball game performance, including decision-making and skill execution (13). Structured cooperative game models and team-based tournaments have similarly enhanced football and futsal performance outcomes by embedding technical practice within competitive and socially interactive contexts (14, 15). Such environments encourage adaptive movement solutions, transfer of learning, and sustained learner engagement. Within this review, exergame-based learning was conceptualised as a technology-mediated instructional approach that integrates game elements and, in some implementations, collaborative interaction. Accordingly, exergaming interventions were included only when they were pedagogically structured within physical education settings and explicitly designed to support movement learning outcomes.

Beyond sport-specific skills, several studies emphasize the broader construct of motor competence and movement fluency as critical educational outcomes. Collaborative game-based interventions have demonstrated improvements in coordination, agility, and integrated movement performance among school and university students (16–18). These findings suggest that pedagogical designs emphasizing cooperation, exploration, and contextualized movement may foster holistic motor development rather than narrowly defined technical proficiency. Collectively, the literature indicates promising educational value across diverse instructional models and learner populations.

Despite increasing empirical attention, evidence on collaborative and game-based pedagogies in physical education remains dispersed across educational levels, instructional frameworks, outcome domains, and levels of methodological rigour. Existing studies predominantly focus on sport-specific technical skills, while comparatively fewer investigations examine fundamental movement skills, tactical game performance, or issues related to learning retention and transfer. Furthermore, variability in study design, intervention duration, and outcome measurement approaches, often coupled with inconsistent use of validated instruments, limits comparability across studies. The concentration of research within university-level populations further constrains the extent to which findings can be generalised to primary and secondary school contexts.

Given the heterogeneity of pedagogical models, participant characteristics, methodological designs, and assessed outcomes, research on collaborative and game-based pedagogies in physical education remains fragmented. Although these approaches are increasingly implemented in practice, variation in instructional formats and assessment strategies limits direct comparison across studies and hinders identification of consistent research patterns. In this context, a systematic review is warranted to provide a structured synthesis of quasi-experimental evidence in formal physical education settings. Accordingly, this review aimed to map existing studies implementing collaborative or game-based pedagogical interventions and assessing movement-related outcomes. Guided by the PICO framework, the review examined the educational levels and participant characteristics represented (P), the pedagogical models employed (I), instructional or grouping comparisons where applicable (C), and the movement-related outcome domains assessed (O), including fundamental movement skills, motor competence, sport-specific skills, and game performance.

Accordingly, this systematic review sought to address the following research question: How have collaborative and game-based pedagogical interventions been implemented and evaluated in quasi-experimental studies conducted in formal physical education settings with respect to movement-related outcomes? The objective of the review was to systematically map quasi-experimental evidence by examining the pedagogical approaches employed, the educational contexts and participant characteristics represented, the movement-related outcome domains assessed, and the overall direction of reported effects. In addition, the methodological quality of the included studies was appraised to identify prevailing methodological patterns and gaps within the existing literature.

## 2 Materials and methods

### 2.1 Study design

This study consists of a systematic review conducted in accordance with the guidelines established by Page et al. (19), known as the Preferred Reporting Items for Systematic Reviews and Meta-Analyses (PRISMA 2020) (19), and registered in the Prospective International Register of Systematic Reviews (PROSPERO) under number CRD420261306696. To ensure transparency, reproducibility, and standardization in the presentation of methods and results (20).

Although this review did not aim to quantitatively estimate the pooled effects of the intervention, it followed a systematic review methodology with predefined eligibility criteria, search strategies, and analytical procedures established prior to study selection. No formal protocol registration was performed; however, all methodological decisions were specified *a priori* to reduce the risk of selection bias and subsequent methodological modification.

### 2.2 Literature search: administration and update

**Figure 1:**
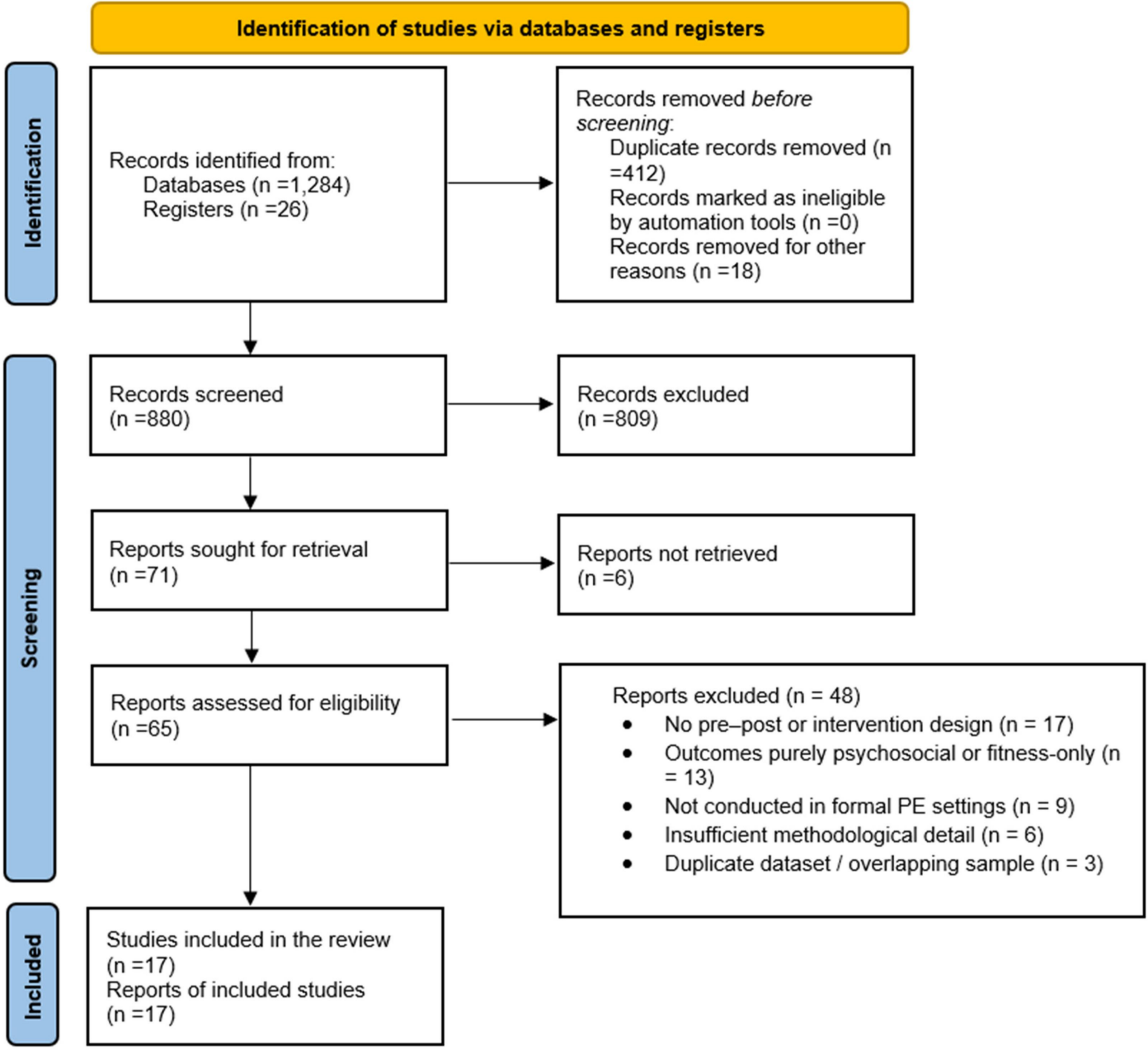
PRISMA 2020 flow diagram illustrating the study selection process.

A comprehensive literature search was conducted across Scopus, Web of Science, PubMed, ERIC, and SPORTDiscus using structured systematic search strategies that combined keywords and subject headings related to collaborative learning, cooperative learning, peer teaching, game-based learning, tactical games, exergaming, physical education, and movement or motor skills. Searches were designed to identify all eligible studies published between January 2011 to December 2025. Additionally, the reference lists of the included studies were systematically examined through manual searching to identify potentially eligible studies that were not retrieved through the electronic database searches. Search strategies were adapted to the indexing and syntax requirements of each database to maximize sensitivity and ensure comprehensive coverage of the literature. After the screening and eligibility assessment process, a total of 17 studies (Figure 1) met the inclusion criteria and were included in the final systematic review.

### 2.3 Eligibility criteria

In summary, the predefined inclusion and exclusion criteria were structured according to the PICOS model. The population was restricted to participants involved in formal contexts of Physical Education or Physical Education Teacher Training, in order to ensure pedagogical and contextual relevance. Eligible interventions were limited to collaborative and game-based pedagogical models that were clearly defined and integrated into Physical Education teaching, thus excluding programs focused exclusively on physical conditioning or non-pedagogical programs (See Table 1).

**Table 1:** Inclusion and exclusion criteria for study selection.

PICOS component	Inclusion criteria	Exclusion criteria
Population (P)	Participants enrolled in formal Physical Education (PE) or PE-related instructional contexts, including preschool, primary, secondary, university, or Physical Education Teacher Education (PETE) programmes	Participants from non-PE contexts, such as sport clubs, elite sport academies, recreational programmes, clinical rehabilitation, fitness centres, or home-based interventions
	Typically developing children, adolescents, young adults, or pre-service PE teachers	Populations with clinical conditions, disabilities, or medical rehabilitation focus where PE instruction was not the primary context
Intervention (I)	Pedagogically structured collaborative or game-based instructional models implemented within PE or PETE, including cooperative learning, peer teaching, Team Games Tournament (TGT), Tactical Games Model (TGM), structured game-based learning, or pedagogically guided exergaming	Interventions focused solely on fitness training, conditioning, or isolated drills without an explicit pedagogical framework
	Interventions embedded within PE lessons, PE curricula, or PE-related coursework	Technology-only interventions (e.g., exergaming, apps) without instructional design or pedagogical intent
Comparator (C)	Traditional PE instruction (e.g., direct instruction), alternative pedagogical models, different grouping strategies, usual practice, or no-intervention control	Studies lacking any comparator and without pre-post outcome assessment
	One-group pre-post intervention studies with clearly described pedagogical implementation and movement-related outcomes (retained for descriptive evidence mapping)	Purely descriptive, correlational, or cross-sectional studies without an intervention
Outcomes (O)	Movement-related outcomes, including sport-specific technical skills, game performance/tactical execution, fundamental movement skills, or general motor competence	Studies reporting only cognitive, academic, emotional, or motivational outcomes with no movement, performance, or movement-derived assessment
	Psychosocial (e.g., motivation, enjoyment, social interaction) or fitness outcomes only when clearly derived from PE participation and pedagogical implementation	Studies measuring only physical activity volume (e.g., MVPA, steps) or fitness variables without pedagogical or movement-learning relevance
Study Design (S)	Quasi-experimental designs, cluster-assigned studies, non-randomised controlled studies, and one-group pre-post intervention studies conducted in PE or PETE contexts	Randomised laboratory-based experiments conducted outside PE settings; qualitative-only studies; purely observational studies without intervention
	Peer-reviewed journal articles published in English	Conference abstracts, theses, dissertations, book chapters, protocols, editorials, and non-peer-reviewed literature

The comparative criteria allowed for both controlled designs and pre-post-test interventions with a single group, provided that a structured pedagogical approach and a pre-post-test evaluation were reported. The outcome criteria prioritized domains related to movement, while psychosocial or physical fitness outcomes were retained only when explicitly derived from participation in pedagogically guided physical education classes. These criteria were established *a priori* and applied uniformly to increase transparency, reproducibility, and methodological rigor in the review process.

### 2.4 Study selection procedures

All records retrieved from electronic database searches were imported into reference management software, where duplicate citations were identified and removed before screening. Two reviewers independently screened titles and abstracts to assess preliminary eligibility according to predefined inclusion and exclusion criteria. Full-text articles of potentially relevant records were subsequently retrieved and independently assessed for final inclusion. Any disagreements arising during the screening or eligibility assessment phases were resolved through discussion and consensus, ensuring consistency and methodological rigour in the selection process. The complete study selection procedure was documented using a PRISMA 2020 flow diagram, transparently reporting the number of records identified, screened, excluded, and included, in accordance with established systematic review reporting guidelines (20, 21).

### 2.5 Data extraction

Data extraction was undertaken using a standardized data charting form developed specifically for this systematic review, following established guidance for structured evidence synthesis (22). The extracted data included bibliographic information, country of origin, study design, participant characteristics and educational level, intervention characteristics, comparator conditions, intervention duration, movement-related outcome domains assessed, outcome measurement instruments, and the direction of reported motor skill outcomes, in accordance with recommended systematic review practices (20, 21). Data extraction was performed independently by two reviewers to enhance accuracy, reliability, and methodological consistency. Any discrepancies identified during the extraction process were resolved through discussion and consensus, consistent with recommended systematic review procedures (19). Where necessary, corresponding authors of the included studies were contacted to clarify missing, incomplete, or ambiguous information, in line with best-practice guidance for transparent and rigorous evidence synthesis (23).

### 2.6 Methodological quality of the included studies

Methodological quality the risk of bias was evaluated using an adapted version of the Joanna Briggs Institute (JBI) Critical Appraisal Checklist for Quasi-Experimental Studies (24). The appraisal addressed key methodological domains, including baseline group comparability, participant selection procedures, clarity and fidelity of intervention delivery, integrity of the control condition, validity and reliability of outcome measurement instruments, completeness of outcome data, appropriateness of statistical analyses, and the potential for selective reporting, consistent with established critical appraisal frameworks (21). Each appraisal domain was rated as representing low, moderate, high, or unclear risk of bias, enabling structured judgments regarding internal validity and transparency of reporting (23, 25). Overall methodological quality was determined by considering the balance of domain-level judgments rather than by calculating a single summary score, in accordance with best-practice guidance for evidence synthesis (19). Quality appraisal was conducted independently by two reviewers to enhance objectivity and methodological rigor, with any disagreements resolved through discussion and consensus (20, 21).

### 2.7 Summary measures

Given the primarily descriptive objectives of this systematic review and the anticipated heterogeneity across study designs, participant characteristics, pedagogical interventions, and outcome measures, data synthesis was descriptive rather than quantitative (20, 21). Frequencies and distributions were used to summarise study characteristics, participant demographics, intervention duration, pedagogical models, outcome measurement instruments, and the direction of reported movement-related effects, consistent with recommended approaches for systematic review synthesis in the absence of meta-analysis (26, 27). No pooled effect sizes, meta-analytic models, or quantitative synthesis metrics were calculated, as the primary objective of the review was to systematically characterise the extent, nature, and methodological features of the available evidence rather than to estimate comparative intervention effectiveness (19, 28).

### 2.8 Synthesis of results

Results were synthesized using a narrative synthesis supported by structured descriptive evidence mapping. Structured tables were employed to systematically summarise key study characteristics, methodological quality, participant distributions, intervention features, outcome measurement strategies, and the direction of reported motor skill effects, in line with recommended approaches for organizing and presenting heterogeneous evidence within systematic reviews (26).

Patterns and trends across educational levels, pedagogical models, and intervention durations were examined to identify areas of consistency, research concentration, and gaps within the existing literature (24). This synthesis approach enabled a comprehensive and transparent characterization of the evidence base while avoiding assumptions of statistical homogeneity or effect-size aggregation, which were not appropriate given the diversity of study designs and outcome measures and were not objectives of the present systematic review (19).

### 2.9 Publication bias

Formal assessment of publication bias using funnel plots or statistical tests was not conducted, as such methods require pooled quantitative effect estimates and are therefore not appropriate for systematic reviews that do not include meta-analysis and do not aim to estimate intervention effects (19–21). Nevertheless, the potential influence of publication bias was mitigated through comprehensive searching across multiple electronic databases, manual screening of reference lists, and inclusion of studies from diverse geographic regions and educational contexts, consistent with recommended strategies to enhance retrieval completeness and reduce selection bias in evidence synthesis (26). Despite these measures, the possibility that studies reporting non-significant or null findings remain unpublished is acknowledged as an inherent methodological limitation (23).

### 2.10 Additional analyses

Additional exploratory analyses were conducted to examine the distribution of included studies by participant age group, educational level, intervention duration, pedagogical model, type of outcome measurement instrument, and methodological quality category. These analyses were descriptive in nature and were undertaken to identify research concentrations, methodological patterns, and underrepresented areas within the literature, thereby informing future research priorities rather than supporting causal inference or comparative effectiveness conclusions (29).

## 3 Results

The results of this systematic review synthesise evidence from 17 studies examining the effects of collaborative, cooperative, peer-mediated, and game-based pedagogical approaches implemented within physical education settings. The included studies span preschool, school, and university contexts, reflecting a wide range of participant ages, instructional designs, and intervention durations. Considerable heterogeneity was observed in study designs, pedagogical frameworks, and outcome measurement instruments, justifying a descriptive and narrative synthesis approach. Despite this variability, clear patterns emerged in relation to pedagogical application and targeted outcome domains. The results are organised to present study characteristics, distributions across educational levels and pedagogical models, outcome domains, and methodological quality. Together, these findings provide a structured overview of the existing evidence while highlighting methodological concentrations and gaps within the literature.

Table 2 presents a comprehensive overview of the characteristics and key findings of the 17 studies included in the review, encompassing diverse educational levels from preschool to university and PETE contexts. The table highlights the range of collaborative and game-based pedagogical models implemented within Physical Education, including peer teaching, cooperative learning, tactical models, and exergaming. Study designs were predominantly quasi-experimental, with a smaller number of one-group pre-post and observational descriptive studies retained for evidence mapping purposes.

**Table 2:** Characteristics of studies (*n* = 17).

Study (Year)	Country	Design/educational level	Participants	Pedagogical intervention	Comparator	Duration	Primary outcome domain	Outcome measures	Key findings
Iserbyt et al. (7)	Belgium	Quasi-experimental; Secondary PE	*n* = 55	Reciprocal peer teaching (task cards; tennis)	Teacher-led instruction	4 weeks	Sport-specific motor skill	Foreground Tennis Test; video analysis	Peer teaching is comparable to teacher instruction
Whipp et al. (8)	Australia	Cluster quasi-experimental; Secondary PE	*n* = 200 (motor *n* = 22)	Trained non-reciprocal peer teaching (TGA)	Untrained peer teaching	5 weeks	Game performance	GPAI; ALT-PE	Large motor learning gains
Hulteen et al. (30)	Australia	Observational descriptive; Elementary	*n* = 19	Active video games	Within-subject	6 weeks	Movement behaviour	OTAGM; TGMD-3	No motor learning
Huang et al. (9)	Taiwan	Cluster quasi-experimental; Elementary PE	*n* = 170	Cooperative Learning & Concept Mapping	Practice Style	15 weeks	Sport skills & cognition	AAHPERD; CT Test	CL & CM > control
Gao et al. (31)	USA	Cluster quasi-experimental; Preschool	*n* = 56	Exergaming	Recess	8 weeks	PA (primary); motor (secondary)	Accelerometry; TGMD-2	PA ↑; no motor advantage
Ye et al. (32)	USA	Quasi-experimental; Elementary PE	n ≈ 250	Exergaming + PE	Traditional PE	9 months	Motor competence & fitness	MSC tests; FITNESSGRAM	Fitness ↑; OC favoured PE
Yang et al. (10)	China	Quasi-experimental; Junior High PE	*n* = 59	Cooperative Learning (grouping comparison)	Cooperative Learning	6 weeks	Sport skills & motivation	Action Skills; ARCS	No grouping effect
Engels & Freund (33)	Germany	Quasi-experimental; School PE	*n* = 285	Cooperative games	Regular PE	7–14 weeks	Psychosocial	QUAEPE	Psychosocial ↑ only
Fizi et al. (16)	Indonesia	One-group pre-post; Elementary PE	*n* = 46	Game-based PE model	None	8 sessions	Motor skills	Speed, agility, balance	Pre-post gains
Rubiyatno et al. (14)	Indonesia	One-group pre-post; Junior High PE	*n* = 31	TGT (football shooting)	None	Single unit	Sport skill	Bobby Charlton Test	Pre-post gains
Kumar et al. (18)	India	Quasi-experimental; University PE	*n* = 60	Cooperative Game-Based Learning	Traditional PE	12 weeks	Motor competence	MCAP	Large motor gains
Longakit et al. (17)	Philippines	Quasi-experimental; College PE	*n* = 60	Game skill-based learning	Conventional PE	12 weeks	Motor competence	MCS	EG > CG
Wang et al. (5)	China	Quasi-experimental; University PE	*n* = 164	Tactical Games Model	Direct instruction	8 weeks	Motivation	SMS-II; SDI	Motivation ↑
Wardhani et al. (34)	Indonesia	Quasi-experimental; Junior High PE	*n* = 62	Game-Based Learning	Conventional PE	8 weeks	Fitness & psychosocial	SPS; fitness tests	Fitness & social ↑
Ayşe (35)	Turkey	Quasi-experimental; University PE	*n* = 70	Peer teaching & CWPT-PE	Direct instruction	12 weeks	Game performance & cognition	GPAI; skill tests	Game performance ↑
Beseler et al. (36)	Australia	Quasi-experimental; University PETE	*n* = 47 pre-service PE teachers (24 males, 23 females); Mage = 20.6 ± 3.4 yrs	Reciprocal peer-teaching of non-dominant overarm throwing; Video Analysis Group (VAG) vs. Verbal Group (VG)	Control group completing unrelated coursework	Single 20-min session; retention at 3 weeks	Fundamental movement skill (throwing technique—process-based)	Roberton developmental levels (6 components); QMD questionnaire	VAG showed significant immediate improvement post-test; no significant between-group differences at retention; single-session effect only; retained for pedagogical and motor-learning mapping
Hamsyah et al. (15)	Indonesia	One-group pre-post; Elementary PE	*n* = 10	TGT (futsal)	None	8 weeks	Sport skill	Vernon Crew Test	Pre-post gains

PE, physical education; PETE, physical education teacher education; TGA, tactical games approach; TGT, team games tournament; PA, physical activity; GPAI, game performance assessment instrument; ALT-PE, academic learning time in physical education; OTAGM, observation tool for active gaming and movement; TGMD, test of gross motor development; MSC, motor skill competence; CT, critical thinking; ARCS, attention-relevance-confidence-satisfaction; MCAP, movement competency assessment protocol; MCS, movement competency screening; SMS-II, sport motivation scale-II; SDI, self-determination index; SPS, social provisions scale; QMD, qualitative movement diagnosis.

Outcome domains varied considerably, spanning sport-specific motor skills, general motor competence, game performance, motivation, psychosocial outcomes, and physical fitness. Measurement approaches included validated motor skill tests, game performance instruments, fitness batteries, and psychosocial scales. Overall, the table illustrates substantial heterogeneity in design and outcomes, while indicating generally positive trends for collaborative and game-based pedagogies, particularly when movement-related outcomes were explicitly targeted.

Table 3 presents the risk of bias assessment of the 17 included studies using key domains relevant to non-randomized and quasi-experimental designs. Most studies demonstrated low to moderate risk of bias, particularly in domains related to intervention delivery and outcome measurement, reflecting acceptable methodological rigor in applied PE settings.

**Table 3:** Risk of bias assessment of included studies (*n* = 17).

Study (Author, year)	D1: randomization	D2: deviations from intervention	D3: missing data	D4: outcome measurement	D5: selective reporting	Overall risk of bias
Iserbyt et al. (7)	Moderate	Low	Low	Low	Low	Low
Whipp et al. (8)	Low	Low	Low	Low	Low	Low
Hulteen et al. (30)	Moderate	Low	Low	Moderate	Low	Moderate
Huang et al. (9)	Moderate	Low	Low	Low	Low	Moderate
Gao et al. (31)	Low	Low	Low	Low	Low	Low
Ye et al. (32)	Low	Low	Moderate	Low	Low	Low
Yang et al. (10)	Moderate	Low	Low	Low	Low	Moderate
Engels & Freund (33)	Low	Low	Low	Low	Low	Low
Fizi et al. (16)	High	Low	Low	Moderate	Moderate	High
Rubiyatno et al. (14)	High	Moderate	Unclear	Moderate	Moderate	High
Kumar et al. (18)	Moderate	Low	Low	Low	Low	Moderate
Longakit et al. (17)	Moderate	Low	Low	Low	Low	Moderate
Wang et al. (5)	Low	Low	Low	Low	Low	Low
Wardhani et al. (34)	Moderate	Low	Low	Moderate	Low	Moderate
Ayşe (35)	Moderate	Low	Low	Moderate	Low	Moderate
Beseler et al. (36)	Low	Low	Low	Low	Low	Low
Hamsyah et al. (15)	High	Moderate	Unclear	Moderate	Moderate	High

D1 = Bias due to randomization or group allocation procedures; D2 = Bias due to deviations from intended intervention; D3 = Bias due to missing outcome data; D4 = Bias in outcome measurement; D5 = Bias due to selective outcome reporting; PE, physical education; PETE, physical education teacher education.

Higher risk ratings were primarily observed in one-group pre-post designs, largely due to the absence of randomization and limited control of confounding variables. Observational and psychosocial-focused studies were retained for descriptive and contextual evidence mapping rather than causal inference. Overall, the evidence base shows reasonable internal validity, with variability aligned to study design rather than systematic methodological flaws.

Table 4 summarises the distribution of the 17 included studies across educational levels, pedagogical models, and primary outcome domains. Nearly half of the studies were conducted in university, college, or PETE contexts, while preschool settings were minimally represented, indicating a strong emphasis on older learners in the existing literature. Pedagogically, collaborative and game-based approaches were evenly distributed across cooperative learning, peer teaching, Team Games Tournament, exergaming, and structured game-based movement models, reflecting methodological diversity rather than dominance of a single instructional approach.

**Table 4:** Distribution of included studies by educational level, pedagogical model, and primary outcome domain (*n* = 17).

Analytical dimension	Category	Studies (*n*, %)	Included studies
Educational Level	Preschool	1 (5.9%)	Gao et al. (31)
	Primary School	4 (23.5%)	Huang et al. (9); Fizi et al. (16); Rubiyatno et al. (14); Hamsyah et al. (15)
	Secondary School	4 (23.5%)	Iserbyt et al. (7); Whipp et al. (8); Yang et al. (10); Wardhani et al. (34)
	University/College/PETE	8 (47.1%)	Ayşe (35); Gao et al. (31); Kumar et al. (18); Longakit et al. (17); Wang et al. (5); Engels & Freund (33); Beseler et al. (36); Luo et al. (12)
Pedagogical Model	Cooperative Learning (incl. CL, CM, grouping variants)	3 (17.6%)	Huang et al. (9); Yang et al. (10); Engels & Freund (33)
	Peer Teaching/Peer-Mediated	3 (17.6%)	Iserbyt et al. (7); Whipp et al. (8); Beseler et al. (36)
	Team Games Tournament (TGT)	3 (17.6%)	Luo et al. (12); Rubiyatno et al. (14); Hamsyah et al. (15)
	Tactical/Games-Based Models (TGA/TGM)	2 (11.8%)	Whipp et al. (8); Wang et al. (5)
	Exergame-Based Learning	3 (17.6%)	Gao et al. (31); Ye et al. (32); Hulteen et al. (30)
	Structured Game-Based Movement Models	3 (17.6%)	Kumar et al. (18); Longakit et al. (17); Fizi et al. (16)
Primary Outcome Domain	Sport-Specific Technical Skills	6 (35.3%)	Iserbyt et al. (7); Ayşe (35); Yang et al. (10); Rubiyatno et al. (14); Hamsyah et al. (15); Huang et al. (9)
	Fundamental Movement Skills/Technique	3 (17.6%)	Hulteen et al. (30); Beseler et al. (36); Ye et al. (32)
	General Motor Competence/Movement Fluency	3 (17.6%)	Kumar et al. (18); Longakit et al. (17); Fizi et al. (16)
	Game Performance/Tactical Outcomes	2 (11.8%)	Whipp et al. (8); Ayşe (35)
	Psychosocial/Fitness-Derived Outcomes	3 (17.6%)	Engels & Freund (33); Wang et al. (5); Wardhani et al. (33)

Sport-specific technical skills constituted the most frequently assessed outcome domain, followed by fundamental movement skills and general motor competence. In contrast, game performance and psychosocial or fitness-derived outcomes were examined less often, highlighting clear gaps for future research in tactical and holistic movement outcomes within physical education.

Table 5 illustrates the distribution of pedagogical models and primary outcome domains across the 17 included studies. Sport-specific technical skills were the most frequently assessed outcomes, followed by fundamental movement skills and general motor competence, while game performance and psychosocial or fitness outcomes were less commonly examined.

**Table 5:** Distribution of pedagogical models and outcome domains (*n* = 17).

Category type	Category	Studies (n)	Representative studies
Outcome Domain	Sport-specific skills	6	Iserbyt (7); Huang (9); Rubiyatno (14)
	Fundamental movement skills	3	Hulteen (30); Beseler (36)
	General motor competence	3	Kumar et al. (18); Longakit (17)
	Game performance	2	Whipp (8); Ayşe (35)
	Psychosocial/fitness	3	Engels & Freund (33); Wardhani (34)
Pedagogical Model	Cooperative learning	3	Huang (9); Yang (10)
	Peer teaching	3	Iserbyt (7); Whipp (8)
	TGT	3	Luo (12); Hamsyah (15)
	Tactical/Games models	2	Whipp (8); Wang (5)
	Exergaming	3	Gao (31); Ye (32)
	Structured game-based	3	Kumar et al. (18); Fizi (16)

From a pedagogical perspective, cooperative learning, peer teaching, and Team Games Tournament (TGT) models were equally represented, alongside structured game-based and exergaming approaches. This spread reflects both the methodological diversity of collaborative pedagogies in physical education and the continued emphasis on sport-specific skill development over broader motor or psychosocial outcomes.

Table 6 integrates participant characteristics, intervention duration, and outcome trends across the 17 included studies to provide an overall synthesis of the evidence base. Most investigations were conducted in university or PETE contexts, while preschool populations were minimally represented, indicating an age-related research imbalance. The majority of interventions were medium-term in duration, reflecting typical curricular implementation within physical education settings.

**Table 6:** Summary of participant characteristics, intervention duration, and outcome trends across included studies (*n* = 17).

Analytical dimension	Category	Studies (n)	Representative examples
Educational Level	Preschool	1	Gao et al. (31)
	Primary School	4	Huang et al. (9); Fizi et al. (16)
	Secondary School	4	Iserbyt et al. (7); Whipp et al. (8)
	University/PETE	8	Kumar et al. (18); Ayşe (35)
Intervention Duration	Single-session	1	Beseler et al. (36)
	Short-term (2–6 weeks)	3	Whipp et al. (8); Yang et al. (10)
	Medium-term (7–12 weeks)	9	Luo et al. (12); Longakit et al. (17)
	Long-term (≥1 academic term)	4	Ye et al. (32); Gao et al. (31)
Direction of Outcome Trends	Significant motor/game-performance improvement	7	Whipp et al. (8); Kumar et al. (18)
	Partial/mixed improvement	5	Luo et al. (12); Ye et al. (32); Beseler et al. (36)
	No motor skill advantage	5	Hulteen et al. (30); Engels & Freund (33)
	Negative effects	0	—

Outcome trends revealed that fewer than half of the studies demonstrated clear motor or game-performance improvements, with a comparable number reporting mixed or null motor effects. Importantly, no study reported negative motor outcomes following collaborative or game-based interventions. This pattern underscores the pedagogical promise of such models while highlighting variability in motor-learning effectiveness depending on context, duration, and outcome focus.

## 4 Discussion

### 4.1 Overview of the evidence landscape

This systematic review mapped evidence from 17 intervention-based studies examining collaborative and game-based pedagogical approaches in physical education and their associations with movement-related outcomes across preschool, school, and university settings. In line with systematic review methodology, the objective was not to determine pooled effectiveness but to desystematic reviewibe patterns, gaps, and methodological characteristics of the literature (20, 21, 26, 27). Overall, the evidence indicates that learner-centred pedagogies grounded in social interaction and game-based learning are frequently associated with improvements in movement competence, sport-specific skills, or game performance, although the magnitude and consistency of effects vary substantially across contexts. Importantly, no study reported detrimental effects on movement outcomes, suggesting that such pedagogical models are at minimum safe alternatives to traditional instruction. However, heterogeneity in intervention duration, instructional fidelity, outcome instruments, and participant age limits direct comparison across studies and reinforces the appropriateness of desystematic reviewiptive evidence mapping rather than comparative synthesis (19, 29).

### 4.2 Effects on motor skill domains

Sport-specific technical skills constituted the most commonly examined outcome domain, particularly in basketball, football, futsal, tennis, and volleyball contexts. Cooperative learning, peer-mediated instruction, and tactical game-based formats generally demonstrated improvements in execution accuracy, movement consistency, and performance efficiency (7, 9, 13, 14). These findings align with ecological and constraints-led perspectives suggesting that representative learning environments enhance perception–action coupling and transfer to authentic performance situations. In contrast, fundamental movement skills were primarily assessed in exergame-based interventions, where improvements in balance, coordination, locomotion, and object control were commonly reported, especially among younger children (31, 32, 37). Nevertheless, evidence for transfer from digital environments to complex sport skills remains mixed, with some studies reporting limited skill specificity despite gains in physical activity or fitness (30). Studies targeting general motor competence and movement fluency through structured cooperative game circuits showed consistent improvements in integrated movement patterns, supporting the role of whole-body, task-diverse learning environments in developing functional movement capacity (16–18). Collectively, these findings suggest that pedagogical alignment with the targeted motor domain is critical, and no single model appears universally optimal across all movement outcomes.

### 4.3 Influence of pedagogical models

Different pedagogical models appear to facilitate motor learning through distinct mechanisms. Cooperative learning approaches emphasized positive interdependence, shared responsibility, and structured peer interaction, producing simultaneous improvements in skill acquisition and motivational outcomes (9, 10, 33). Peer teaching models promoted reciprocal feedback, observation, and learner autonomy, which were associated with enhanced technique acquisition and performance consistency in sport-specific tasks (7, 36). Team Games Tournament formats integrated competition with cooperative goal structures and were particularly effective for football and futsal passing and shooting performance, although motivational effects often exceeded direct motor gains in more complex tasks (12, 14, 15). Tactical Games Model interventions uniquely targeted tactical awareness and decision-making, demonstrating strong improvements in game performance metrics, particularly at university level (13), consistent with prior syntheses highlighting TGM's strengths in perceptual-cognitive skill development (5). Exergame-based learning primarily enhanced engagement and foundational skills, but evidence of transfer to sport-specific competence remains limited (30, 32). These findings reinforce that pedagogical effectiveness is context-dependent and mediated by instructional design, task representativeness, and learner developmental stage.

### 4.4 Educational level and developmental considerations

The evidence base remains weighted toward university and college populations, where collaborative, peer-mediated, and tactical pedagogies consistently supported engagement, peer interaction, and learning outcomes (8, 13, 18). However, findings derived from university and Physical Education Teacher Education (PETE) contexts should be interpreted cautiously when extrapolating to school-based physical education, as pre-service teacher education aims not only to develop movement competence but also to foster pedagogical knowledge, instructional skills, and teaching effectiveness. Consequently, movement-related outcomes observed in PETE populations are not directly comparable to those reported in children and adolescents. This aligns with broader educational research indicating that peer support mediates engagement and learning effectiveness in higher education settings (11, 38). In primary school contexts, cooperative learning and exergaming interventions demonstrated benefits for both motor development and engagement, supporting the developmental appropriateness of playful, socially interactive learning environments for early skill acquisition (9, 31, 37). By contrast, relatively few studies addressed secondary school populations, despite this being a critical period characterized by declining physical activity participation and increasing skill specialization. The limited and fragmented adolescent evidence highlights an important research gap, particularly given the motivational and social challenges typical of this developmental stage (30, 32, 34).

### 4.5 Measurement practices and outcome sensitivity

Outcome measurement was dominated by sport-specific skill tests and task-based execution metrics, reflecting a traditional emphasis on observable technical proficiency. While such measures are sensitive to short-term performance changes, they may insufficiently capture adaptability, perceptual decision-making, and transfer across contexts. Only two studies employed validated game performance instruments to assess tactical behaviors in authentic game situations (8, 13), despite theoretical emphasis on game-based learning promoting decision quality. Fundamental movement skill batteries were largely confined to exergame research (31, 32, 37), limiting cross-model comparisons of foundational competence. Several studies incorporated psychosocial and fitness indicators, highlighting the multidimensional benefits of collaborative pedagogies (12, 33, 34). Greater integration of standardized movement competence tools, ecological game-performance assessments, and longitudinal retention measures would strengthen future evidence synthesis and theoretical interpretation (1, 39).

### 4.6 Methodological quality and evidence strength

Most included studies demonstrated low-to-moderate risk of bias, particularly those employing structured peer teaching and tactical game models with validated instruments and appropriate statistical analyses (7, 8, 13). Higher risk ratings were associated with single-group pre–post designs, limited baseline equivalence, and unclear attrition reporting, particularly within some cooperative and TGT-based interventions (14–16). Short-duration interventions and single-session designs constrain inference regarding long-term motor learning and retention (36). While formal risk-of-bias assessment is not mandatory in systematic reviews, appraisal supports transparent interpretation of evidence strength (24). The overall convergence of positive findings across diverse contexts suggests pedagogical promise, but caution is warranted when interpreting causal strength.

### 4.7 Practical implications for physical education

The findings support the pedagogical integration of cooperative learning, peer teaching, tactical games, and structured game-based movement models as viable strategies for enhancing movement learning in physical education. Teachers may leverage collaborative structures to promote engagement, feedback exchange, and meaningful practice, particularly in sport skill development contexts (7, 9). Tactical and peer-mediated models appear particularly beneficial for developing decision-making and performance quality in older students (13). Exergaming may serve as a motivational adjunct for younger learners to support foundational movement competence, though it should not replace direct skill instruction (30, 31). Curriculum designers should align pedagogical models with developmental stage, learning objectives, and resource availability to optimize instructional effectiveness. Recent evidence further suggests that hybrid pedagogical approaches combining game-based and learner-centred models may offer particular promise for adolescent populations. For example, combined Teaching Games for Understanding and Sport Education interventions have been shown to improve psychomotor skills alongside emotional and intrapersonal outcomes, while hybrid pedagogical programmes integrating tactical and responsibility-based approaches have demonstrated concurrent improvements in motor and cognitive functions among adolescents (40, 41). These findings reinforce the notion that pedagogical effectiveness may be enhanced when instructional models are purposefully integrated and aligned with multiple learning objectives. This recommendation is consistent with broader evidence suggesting that high-quality physical education is characterized by learner-centred pedagogies that simultaneously foster movement competence and cognitive, affective, and social learning outcomes (42). Taken together, the present evidence does not support the superiority of any single pedagogical model. Rather, the effectiveness of collaborative and game-based approaches appears to depend primarily on the alignment between instructional design, learner characteristics, and the specific movement outcomes being targeted.

### 4.8 Limitations and future research directions

Despite encouraging trends, important gaps remain in longitudinal designs, adolescent populations, standardized outcome measurement, and cross-cultural replication. Future research should examine hybrid pedagogical models that integrate tactical, cooperative, and perceptual–cognitive components, assess retention and transfer of learning, and incorporate biomechanical and decision-making indicators. Expanding research beyond higher education and sport-specific contexts will enhance developmental generalizability and inform inclusive curriculum design. Improved methodological consistency, transparent reporting, and theory-driven intervention design will further strengthen the evidence base supporting collaborative and game-based pedagogies in physical education.

## 5 Conclusions

This systematic review mapped quasi-experimental evidence on collaborative and game-based pedagogical approaches in physical education, synthesising findings from 17 studies across preschool, school, and university contexts. The evidence indicates that learner-centred pedagogies, such as cooperative learning, peer teaching, tactical games, and structured game-based movement models, are frequently associated with positive movement-related outcomes, particularly when instructional design is closely aligned with targeted motor domains. However, effects varied substantially across educational levels, intervention durations, and outcome measures, with fewer than half of studies demonstrating clear motor or game-performance improvements. Methodological heterogeneity, limited adolescent-focused research, and inconsistent use of ecologically valid outcome instruments constrain generalisability and causal interpretation. Overall, collaborative and game-based pedagogies appear to be pedagogically viable and developmentally appropriate alternatives to traditional instruction, but future research should prioritise longitudinal designs, underrepresented age groups, and integrated assessment of motor, tactical, and transfer outcomes to strengthen evidence-informed physical education practice.

## Data Availability

The original contributions presented in the study are included in the article/Supplementary Material, further inquiries can be directed to the corresponding author/s.
